# Anticancer Activity of Liquid Treated with Microwave Plasma-Generated Gas through Macrophage Activation

**DOI:** 10.1155/2020/2946820

**Published:** 2020-01-31

**Authors:** Chae Bok Lee, Il Hwan Seo, Myoung-Won Chae, Jae Woo Park, Eun Ha Choi, Han Sup Uhm, Ku Youn Baik

**Affiliations:** ^1^Department of Medical Science, Chungnam National University, Daejeon 35015, Republic of Korea; ^2^Department of Microbiology, College of Medicine, Chungnam National University, Daejeon 35015, Republic of Korea; ^3^Department of Electrical and Biological Physics, Kwangwoon University, Seoul 01897, Republic of Korea; ^4^Plasma Bioscience Research Center, Kwangwoon University, Seoul 01897, Republic of Korea; ^5^New Industry Convergence Technology R&D Center, Ajou University, Suwon 16499, Republic of Korea

## Abstract

Reactive nitrogen species (RNS), including nitric oxide (NO^·^) has been known as one of the key regulatory molecules in the immune system. In this study, we generated RNS-containing water treated with microwave plasma-generated gas in which the major component was nitric oxide (PGNO), and the effect on the macrophage polarization was investigated. The RNS-containing water was diluted in complete cell culture media (PGNO-solution) into the concentration that did not induce cell death in RAW 264.7 murine macrophages. PGNO-solution upregulates M1-type macrophage activation and downregulates the characteristics of M2-type macrophage at the transcriptional level. In addition, the PGNO-solution-treated M2-like macrophages had higher potential in killing melanoma cells. The anticancer potential was also investigated in a syngeneic mouse model. Our results show that PGNO-solution has the potential to convert the fate of macrophages, suggesting PGNO-solution treatment as a supportive method for controlling the function of macrophages under the tumor microenvironment.

## 1. Introduction

Recently, plasma-activated water (PAW) or media (PAM) has been introduced as an effective solution in killing various kinds of cancer cells, including ovarian cancer, cervical cancer, pancreatic cancer and glioblastoma [1–6]. PAW or PAM has several merits comparing to the direct plasma treatment. PAM does not have any possibility of electrical hazards, it can be stored at low temperature for a certain period, and it can be injected into any part of the body. Many researchers have tried to understand the working mechanisms of PAM in its tumoricidal activity. Since PAM contains relatively long-lived reactive species compared to direct plasma treatment, hydrogen peroxide and nitric oxide ions were identified as the main players in PAM reactivity [7–16]. The actions of reactive oxygen species (ROS) and reactive nitrogen species (RNS) are distinguished in biological systems [17]. In general, ROS induces oxidative stress which can generally activate redox responses in cells; meanwhile, RNS induces nitrosylation or nitrosation which initiates different cellular signaling pathways that are known to be related to cell differentiation or the wound-healing process [18–20]. In this study, we generated PAW that mainly contained RNS and applied them to innate immune cell macrophages, to study their polarized differentiation especially when they interact with cancer cells.

One of the important factors in anticancer treatment is the microenvironment of cancer tissues. It is well known that cancer cells create microenvironments that support their growth and suppress antitumor immune activities. It is typically characterized by low oxygen, low pH, high lactate, high RNS, etc., which results in differentiated responses of cellular components [21]. Monocytes, one of the most prominent components, are recruited in tumor tissues and are polarized to tumor-supporting macrophages, instead of the well-known inflammatory M1 polarized one [22–24]. They are called tumor-associated macrophages (TAMs), which are classified to alternative polarization that is simply called M2 polarization. Since TAMs are known to promote all aspects of tumor initiation, growth, and development and affect the antitumor efficacy of chemotherapy and radiotherapy, converting TAMs to M1 macrophages is a very important issue in cancer therapy.

Though the complex process of programming TAMs has not yet been elucidated, it has been known that immune responses in tumor tissues are tailored by RNS as well as by cytokines, chemokines, or metabolites [25–27]. RNS have shown both immune-suppressive and immune-activate effects in the tumor region in various conditions [27–29]. RNS are mainly synthesized by inducible nitric oxide synthase (iNOS) in response to external stimuli. M1 macrophages express high amounts of iNOS to induce cancer apoptosis; however, the expression of iNOS in TAM was suppressed in tumor microenvironment to maintain low concentration of NO^·^ that is related to angiogenesis or metastasis [25–29]. The expression of iNOS is closely related to the polarization of macrophages, and therefore, the expression of iNOS in macrophage is used as a marker for their anticancer activity [24, 30].

In this study, we generated RNS-containing water treated with microwave plasma-generated gas in which the major component was nitric oxide (PGNO) and investigated its effects on the anticancer effect of macrophages *in vitro* and *in vivo* [31–33]. Pure water was treated with PGNO and the water was diluted in complete media (PGNO-media) for cell study. The proliferation, polarization, and anticancer function of macrophages were examined. Among various kinds of cancer, we chose melanoma, a type of skin cancer, due to the ease of approach as well as its malignancy [34]. It was reported that melanoma also regulates the microenvironment, to let macrophages promote angiogenesis and tumor growth [35]. Murine cell lines Raw 264.7 macrophage and B16F10 melanoma were used for *in vitro* study, and C57BL/6 normal mice were used for *in vivo* study. C57BL/6 has normal immune functions, and the syngeneic models were made by subcutaneously injecting B16F10 cells. To mimic TAM, Raw 264.7 macrophage cells were pretreated with interleukin-4 (IL-4) [36, 37]. The effects of RNS-containing PAW on tumor immunity were discussed.

## 2. Materials and Methods

### 2.1. PGNO-Generating Microwave Plasma

**Figure 1:**
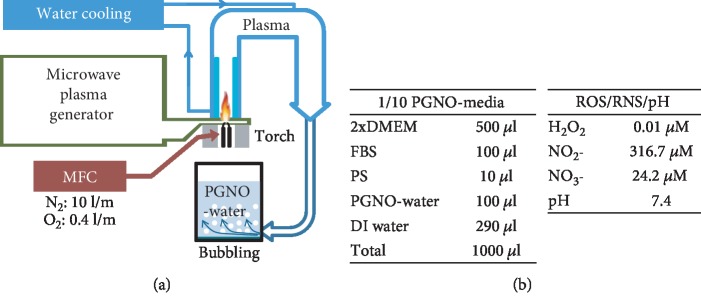
(a) Schematics of the microwave plasma generator and reactor to generate PGNO-water. (b) Composition of 1/10 PGNO-media and its characteristics; concentrations of NO_x_ and H_2_O_2_, and pH.

Figure 1(a) shows a schematic of PGNO-generating microwave plasma. The microwave plasma device consists of power supply, magnetron, waveguide components (WR-340 for 2.45 GHz), and a microwave plasma torch. The microwave radiation from the magnetron passes through the circulator, through the power meter, through the tuner, which tunes the impedance of the plasma, and through the torch. Nitrogen gas enters the discharge tube in the form of a swirling gas through a feeder, which leads to a vortex flow in the discharge tube. The gas flow rate was controlled by mass flow controller, which keeps the flow rate of N_2_ gas 10.0 L per min and O_2_ gas 0.4 L per min. The detailed design and function of the microwave plasma torch system are reported in previous reports [31, 32]. The torch is initiated by an igniter, and 400 W electric power is applied. The heated gas from the torch flame is cooled to room temperature with passing through a water cooling tube, and then, the cooled gas is injected into 1 L deionized (DI) water for 50 min. To reduce the reactions of the cooled gas with dissolved O_2_ in water, DI water is purged with pure N_2_ gas for 1 h, before the plasma ignition. NO radicals generated from the microwave plasma device are dissolved in DI water, and it is diluted with cell culture media (PGNO-media), as shown in Figure 1(b).

### 2.2. Measurement of pH, NO_x_, and H_2_O_2_ in PGNO-Media

The concentration of H_2_O_2_ was determined with Amplex red reagents (A22188, Thermo Fisher Scientific), and the concentrations of *NO*_2_^−^ and *NO*_3_^−^ were measured with a nitric oxide colorimetric assay kit (K262-200, BioVision), following the manufacturer's protocols. All the measurements were made within 5 min from PGNO-media generation and were repeated at least three times. The fluorescence intensity and color changes were measured by a plate reader (Synergy HT, BioTek). The pH was measured by a pH meter (Eutech Instruments, Singapore).

### 2.3. Cell Culture and Viability Assays

Raw 264.7 and B16F10 mouse cell lines were purchased from the Korean cell line bank. Cells were cultured in complete DMEM (LM001-05, Welgene) containing 10% fetal bovine serum (FBS; Biowest) and 1% of antibiotics (LS203-01, Welgene). Cells were maintained in 5% CO_2_ and humidified air at 37°C. To measure the influence of the PGNO-media on cell viability, cells were plated in 96-well plates (SPL30096, SPL or 3610, Corning Inc.), with a density of 5 × 10^3^ per well. Cell viability was measured using the CellTiter 96 Aqueous one solution cell proliferation assay solution (G3582, Promega) and CellTiter-Glo luminescent cell viability assay solution (G7572, Promega) at 1, 2, and 3 days elapsed from PGNO-media treatment. The absorbance at 490 nm and luminescence were measured by a plate reader (Synergy HT, BioTek). Cell morphology was observed by inverted microscopy (Eclipse Ti-U, Nikon).

### 2.4. Flow Cytometry

For intracellular NO^·^ measurement, DAF-FM DA (D23844, Thermo Fisher Scientific) was used. Raw 264.7 cells were seeded in a 6-well plate with a density of 4 × 10^5^ per well. Cells were incubated with 20 ng/mL IL-4 (recombinant mouse IL-4, R&D Systems) for 24 h, and the media was replaced with PGNO-media. After 24 h incubation with PGNO-media, cells were stained with 10 *μ*M DAF-FM DA in DPBS (LB001-02, Welgene) for 20 min at 37°C. After washing 2 times with DPBS, cells were incubated in media for an extra 20 min. Then, cells were detached, and the fluorescent signal per cell was analyzed by a flow cytometer (BD Verse, BD Biosciences). LPS (L4391, Sigma) was used with a concentration of 10 ng/mL, SNAP (S-Nitroso-N-Acetyl-D,L-Penicillamine; Sigmal) was used with a concentration of 50 *μ*M, and cPTIO (2-(4-carboxyphenyl)-4,5-dihydro-4,4,5,5-tetramethyl-1H-imidazolyl-1-oxy-3-oxide, monopotassium salt; Cayman) was used with a concentration of 50 *μ*M.

For staining with antibodies, anti-iNOS-PE (12-5920-80, eBioscience), anti-CD163-PE (bs-2527R-PE, Bioss), and anti-CD86-FITC (bs-1035R-FITC, Bioss) were used. Cells were fixed in 4% paraformaldehyde at 4°C for 10 min. After washing two times with DPBS, cells were permeabilized in 0.05% triton X-100 at RT for 15 minutes. After washing two times with DPBS, cells were blocked with 1% BSA 4°C for 1 hr. Then, they were stained with antibodies (1 : 200) at 4°C for 1 hr. After washing three times with DBPS, fluorescence was analyzed by flow cytometry (BD Verse, BD Biosciences).

For apoptosis analysis, Annexin V-FITC apoptosis detection kit (BD 556547, BD Biosciences) was used. After 24 h incubation with PGNO-media, cells were washed with cold DPBS, resuspended in 1x binding buffer, and then stained with Annexin V-FITC and propidium iodide (PI). After washing with 1x binding buffer, cells were analyzed by flow cytometry (BD Verse, BD Biosciences).

For staining of peritoneal macrophages, Alexa Fluor 594 anti-mouse/human CD11b antibody (101254, BioLegend) and anti-mouse iNOS-PE (12-5920-80, eBioscience) were used. Peritoneal macrophages were harvested from mouse peritoneal and treated with 1 mL RBC lysis buffer at 4°C for 5 min for two times. Cells were fixed in 4% paraformaldehyde at 4°C for 10 min. After washing two times with DPBS, cells were permeabilized in 0.05% triton X-100 at RT for 15 minutes. After washing two times with DPBS, cells were blocked with 1% BSA 4°C for 1 hr. Then, they were stained with CD11b antibody (1 : 200) at 4°C for 1 hr. After washing three times, they were stained with iNOS antibody (1 : 200) at 4°C for 1 hr. After washing three times with DBPS, fluorescence was analyzed by flow cytometry (BD Verse, BD Biosciences).

### 2.5. Measurement of a Transcription Level (qPCR)

Raw 264.7 cells were seeded in a 6-well plate with a density of 4 × 10^5^ per well. Cells were incubated with 20 ng/mL mouse IL-4 from 24 h before PGNO-media stimulus. After 24 h incubation in PGNO-media, cells were harvested and total RNAs were extracted using the RNeasy Mini Kit (Qiagen). The total RNAs were converted to cDNAs using reverse transcriptase and random primers (ReverTra Ace qPCR Master Mix, Toyobo), according to the manufacturer's protocol. The same amount of extracted total RNA taken from each sample was used in cDNA synthesis. The synthesized cDNAs were used in real-time PCR (CFX96TM Real-Time System, Bio-Rad). SYBR was used to quantify the amount of dsDNA. The relative amount of mRNA expression was normalized by that of GAPDH and expressed as a fold change to control. The relative gene expression was evaluated by the comparative cycle-threshold method. The experiments were repeated at least three times. The primer sequences are as follows: NOS2 (F: 5′-GTGGTGACAAGCACATTTGG, R: 5′-AAGGCCAAACACAGCATACC), ARG-1 (F: 5′CGCCTTTCTCAAAAGGACAG, R: 5′GACATCAACAAAGGCCAGGT), IL-6 (F: 5′AGTTGCCTTCTTGGGACTGA, R: 5′TCCACGATTTCCCAGAGAAC), TNF-*α* (F: 5′TGTTGCCTCCTCTTTTGCTT, R: 5′TGGTCACCAAATCAGCGTTA), IL-10 (F: 5′CATGGGTCTTGGGAAGAGAA, R: 5′AACTGGCCACAGTTTTCAGG), CCL17 (F: 5′ACATAAAACGGCCTGTGACC, R: 5′TTTGTGTTCGCCTGTAGTGC), MMP9 (F: 5′AGGTGGACCATGAGGTGAAC, R: 5′CGGTTGAAGCAAAGAAGGAG), EGF (F: GAACAAGAGGACTGGCCAAA, R: 5′ATGGATGGACCACAACCAGT), VEGFA (F: 5′CCAGGAGGACCTTGTGTGAT, R: 5′GGGAAGGGAAGATGAGGAAG), and GAPDH (F: 5′AGAACATCATCCCTGCATCC, R: 5′ACACATTGGGGGTAGGAACA).

### 2.6. Western Blot Analysis

Cells were washed with DPBS, lysed with RIPA lysis buffer (GenDepot, Barker, TX) containing 1% of 100x protease inhibitor cocktail (GenDepot, Barker, TX), and incubated for 30 min on ice. Lysates were centrifuged at 19,000 g for 30 min at 4°C, and the supernatant was mixed with 25% of 4x denaturating buffer (100 mM Tris-HCl, pH 6.8, 4% SDS, and 20% glycerol with bromophenol blue) and heated for 5 min. The proteins were separated through 10% SDS-PAGE gels and were transferred to a nitrocellulose membrane by Mini Trans-Blot Cell (Bio-Rad, CA). The membrane was blocked in 5% BSA in TBS containing 0.1% Tween 20 (TBS-T) for 1 h and incubated overnight with the intended antibodies in and 3% BSA. Excess primary antibodies were then removed by washing with TBS-T for 3 times. The membrane was then incubated with HRP-conjugated secondary antibodies (0.1 *μ*g/mL, anti-rabbit) for 1 h. After three washes with TBS-T, bands were visualized by western blot and exposed to X-ray film, or by ChemiDoc Imaging Systems (Bio-Rad). The original film images were supported in Figs. [Supplementary-material supplementary-material-1] and [Supplementary-material supplementary-material-1] (the supporting information).

### 2.7. Coculture of B16F10 and Raw 264.7 Cells

For in-direct coculture of Raw 264.7 with cancer cells, an insert made of polycarbonate with pores of 0.4 *μ*m in diameter was used in a 24-well plate (37024, SPL). Raw 264.7 cells were cultured on insert membrane with a density of 1 × 10^4^ per well, and B16F10 were cultured in the lower 24-well plate with a density of 2.5 × 10^4^ per well, separately. When cells were fully adhered, cells faced each other with 500 *μ*L and 700 *μ*L media for insert and well plate, respectively. Some of Raw 264.7 cells were pretreated with 20 ng/mL IL-4 from 24 h before 1/10 PGNO-media stimuli, where PGNO-water was diluted 1/10 with complete media.

### 2.8. Subcutaneous Administration of PAW in the Mice

Twelve C57BL/6 mice were purchased from OrientBio in Korea. The animals were fed with sterile and commercial mouse diet and were provided with water *ad libitum*. Animal experiments were approved by the Institutional Research and Ethics Committee at Kwangwoon University (permission number: KWU-PBRC1701004). All the animal experiments were performed in accordance with relevant guidelines and regulations. B16F10 in the concentration of 1 × 10^5^ cells/mL was injected subcutaneously at the right side of the back of mice [38]. Seven days later, mouse hair was shaved for observation. Then, 1 mL of 1/10 PGNO-media or 1x DPBS was administered subcutaneously near the position where the cancer cells were injected every day for the following 12 days. The body weights were measured every day (Fig. [Supplementary-material supplementary-material-1], the supporting information). After 12 days' treatment, mice were euthanized by CO_2_ gas, and the tumor tissues were harvested. The width, length, and height of tumor tissues were measured, and the tissue volumes were calculated with multiplication of those values. Lastly, peritoneal macrophages were harvested with DPBS lush three times. Total 6 mL ice-cold DPBS was poured in the peritoneal cavity and retracted by a syringe. The harvested solution was centrifuged, and the precipitated cells were analyzed for further analysis.

### 2.9. Statistical Analysis

Statistical significance was determined using unpaired Student's *t*-test (two-tail, equal SD). It is considered statistically significant when *p* < 0.01 and *p* < 0.05 (^∗^, *p* < 0.05; †, *p* < 0.01). Means and standard errors were calculated and plotted in the graphs. Analysis was completed using Microsoft Excel.

## 3. Results

### 3.1. Properties of PGNO-Media

Figure 1(a) shows a schematic of the microwave plasma torch that was designed to generate NO^·^ radicals when N_2_ and O_2_ mixture gas was fed into the discharge area. According to the previous reports, we flowed 10 L/min N_2_ gas and 0.4 L/min O_2_ gas through the discharge area, and the plasma was cooled during passing through water-cooling tubes [32]. Finally, NO^·^ containing gas from the microwave plasma passed through 1 L of deionized (DI) water for 50 min, which was previously purged with N_2_ gas for 1 h to expel the dissolved oxygen molecules. The concentration of NO^·^ in this water was measured as 117 *μ*M by an electrochemical method, and it was called PGNO-water [33]. In order to estimate the biological effects of PAW, we diluted PAW in cell culture media as shown in Figure 1(b) and measured the long-lived reactive species, H_2_O_2_ and NO_x_^−^ within 30 min from generation. The concentration of total NO_x_ was measured to be about 340 *μ*M in 1/10 diluted solution. Most of them were in the form of NO_2_^−^, while about 5% were NO_3_^−^. The concentration of H_2_O_2_ was very low and was almost nondetectable. The pH of PGNO-water was initially 2.8, but the value became 7.4 in buffered PGNO-media. Figure 1(b) summarizes in table form the characteristics of 1/10 PGNO-media made for cell studies.

### 3.2. Macrophage Cell Viability after Stimulation with PGNO-Media

**Figure 2:**
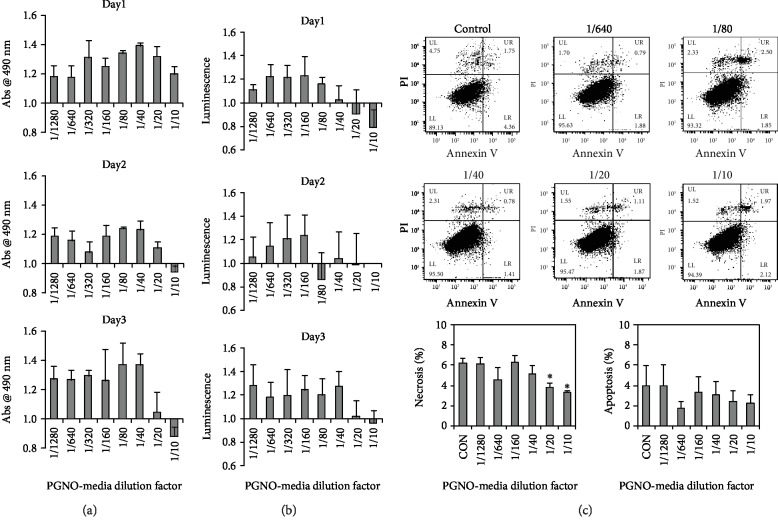
Viability assays of Raw 264.7 macrophages after PGNO-media treatment in various dilution ratios: (a, b) Measurements of MTS and intracellular ATP amount, respectively, at days 1, 2, and 3 elapsed from PGNO-media treatment (*n* = 3). (c) Flow cytometric measurement of Annexin V and PI staining at day 1 elapsed from PGNO-media treatment, and bar graphs of the averaged values for four repetitive experiments. ^∗^*p* < 0.05.

The cytotoxic effects of PGNO-media on Raw 264.7 macrophages were evaluated by measuring the mitochondrial activity and the intracellular ATP amount. Figures 2(a) and 2(b) show each measurement for 3 days that was expressed relative to the control of each day. Both values were enhanced in highly diluted PGNO-media, while both were reduced in slightly diluted PGNO-media. These data support that the dilution more than 20 times was not toxic at all and even enhanced cellular viability. The cytotoxicity of PGNO-media was again confirmed with Annexin V and PI (propidium iodide) staining after 24 hours of incubation in PGNO-media.  Figure 2(c) shows the flow cytometry data whose *x*-axis is the fluorescence intensity of Annexin-V and *y*-axis is the fluorescence intensity of PI. Most cells were plotted in the lower left region, which supports that PGNO-media diluted more than 10 times did not induce apoptosis in macrophages. Interestingly, the ratio of necrotic and apoptotic cells rather decreased in PGNO-media as shown in the averaged bar graphs of four repetitive experiments. It seems that the PGNO-media diluted more than 10 times did not induce apoptosis, though it reduced metabolic activity slightly. In the following studies, we used the same dilution factors to examine the effects of PGNO-media on the physiology of macrophages.

### 3.3. Morphological Changes of Macrophages in PGNO-Media

**Figure 3:**
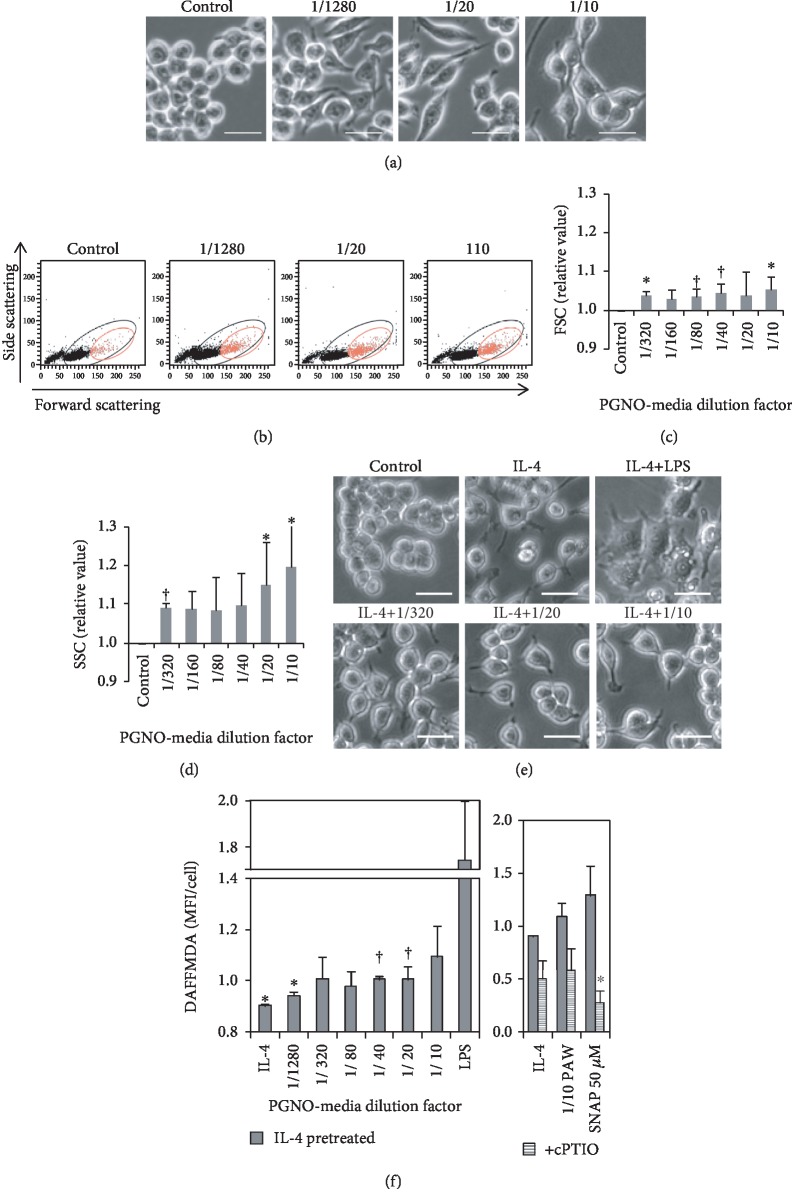
Activation of Raw 264.7 at 24 h elapsed from PGNO-media treatment in various dilution ratios: (a) phase contrast bright field images showing morphological changes. Scale bars are 20 *μ*m. (b) Flow cytometry plot of forward scattering (FSC) and side scattering (SSC) showing morphological changes (*n* = 6). (c, d) Mean values of FSC and SSC ratios, respectively. (e) Phase contrast bright field images of IL-4-pretreated cells for 24 h. Cells were then incubated in LPS and PGNO-media dilutions for 24 h. (f) MFI of DAFFMDA-stained cells that were treated the same as (e) (*n* = 3). IL-4-pretreated macrophages were incubated in PGNO-media or 50 *μ*M SNAP with or without cPTIO for 24 h. ^∗^*p* < 0.05; ^†^*p* < 0.01.

PGNO-media induced morphological changes and size variations in macrophages.  Figure 3(a) shows the phase-contrasted bright field images of Raw 264.7 cells that were incubated in PGNO-media for 24 h. Some round macrophages became somewhat fibroblastic when stimulated with PGNO-media. The morphological changes were confirmed statistically using flow cytometry.  Figure 3(b) shows the plots of forward scattering (FSC) and side scattering (SSC) light intensity of cells in each group. It is clear that the ratio of red spots increased, where the mean values of FSC and SSC were higher. The increase of FSC value implies the increase of cell size, and the increase of SSC implies the increase of surface roughness or internal granularity. The data are plotted as bar graphs in Figures 3(c) and 3(d), which show that the mean values of FSC and SSC of macrophages increased together with the dilution factor. These data imply that PGNO-media induced morphological changes and size variation of macrophages. In order to mimic TAM, cells were pretreated with IL-4 for 24 h, and the medium was changed to PGNO-media, LPS (lipopolysaccharides), or cell culture media.  Figure 3(e) shows the phase contrast bright field images of IL-4-pretreated cells. Compared to small and round control cells, IL-4-treated cells became more flattened and elongated. PGNO-media-treated cells did not induce more morphological changes as LPS that made cells be severely deformed.

### 3.4. Increase of the Intracellular NO^·^ inside the Macrophage in PGNO-Media

The intracellular NO^·^ has been intensively studied as a main indicator and as an important factor for macrophage activation [20]. Intracellular NO^·^ was stained with DAFFMDA, and the fluorescence intensity per cell was measured by flow cytometry after 24 h of incubation with PGNO-media. In order to check an intracellular NO^·^ increase in polarized macrophages like TAM, Raw 264.7 cells were preincubated with IL-4 for 24 h.  Figure 3(f) shows the mean fluorescence intensities (MFI) per cell, which were expressed as relative values to control. The first bar value is significantly lower than control, which represents the significant reduction of the intracellular NO when macrophages were incubated with IL-4. However, it is clear that PGNO-media enhanced the amount of intracellular NO^·^ in IL-4-treated M2-like macrophages. The change of intracellular NO^·^ could be related to the iNOS expression, which is a regulatory molecule in macrophage polarization. These changes in intracellular NO^·^, as well as cell morphology, suggest possible regulatory effects of our PGNO-media on macrophage activations. In order to confirm whether the enhanced values were intracellular NO^·^, we used well-known NO^·^ scavenger (cPTIO) and the well-known NO^·^ donor (SNAP). The intracellular NO^·^ level increased with the addition of SNAP as with PGNO-media, and the levels decreased with the addition of cPTIO into similar levels to control.

### 3.5. Action of PGNO-Media on Repolarization of M2-Like Macrophage

**Figure 4:**
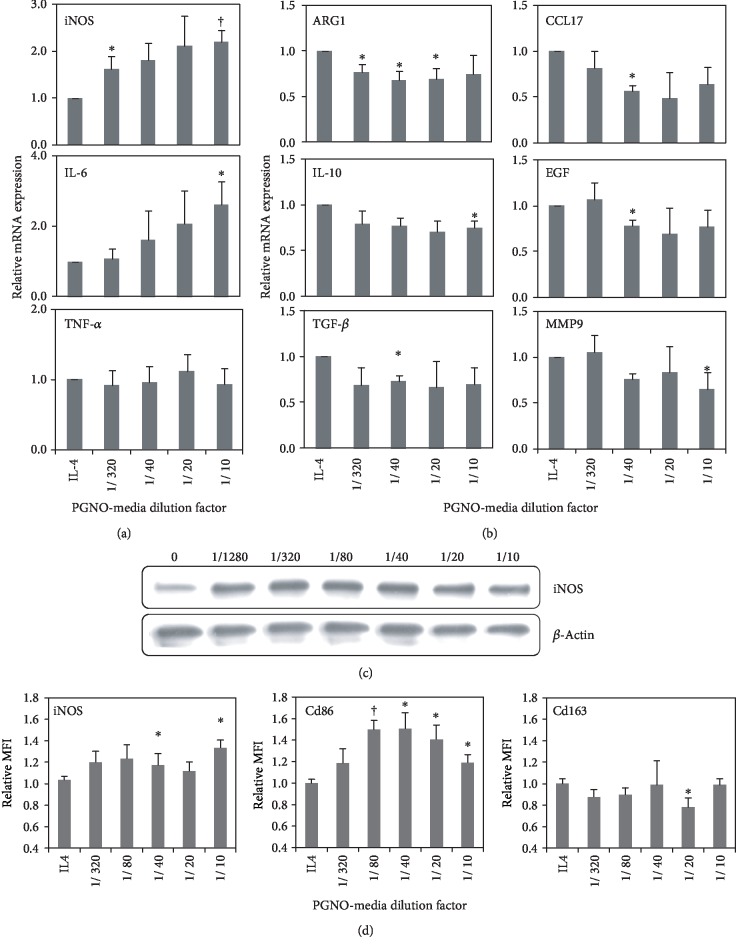
Depolarization of IL-4-pretreated Raw 264.7 macrophages at 24 h elapsed from PGNO-media treatment in various dilution ratios: (a, b) transcriptional level changes of macrophage M1 polarization-related genes, (a) including iNOS, IL6, and TNF-*α*, and (b) M2 polarization-related genes, including ARG, IL10, TGF-*β*, CCL17, EGF, and MMP9. (c) Protein level changes of iNOS. All tests were repeated three times. (^∗^*p* < 0.05; ^†^*p* < 0.01). (d) Flow cytometry analysis of iNOS, CD86, and CD163 proteins.

In order to examine the effect of PGNO-media on the polarization of M2-like macrophages, we analyzed the expression levels of M1- or M2-related proteins in mRNA levels in IL-4-pretreated macrophages after 24 h of incubation in PGNO-media. The mRNA expression of M1-polarization-related proteins, such as iNOS and IL-6, increased according to PGNO-media dilution factor, but the increase of TNF-*α* was not significant (Figure 4(a)). On the other hand, the mRNA expression of M2-polarization-related proteins, such as ARG1, IL-10, TGF-*β*, CCL17, EGF, and MMP9, was mostly reduced significantly (Figure 4(b)). The expression of iNOS was further examined at a protein level.  Figure 4(c) shows the upregulation of iNOS in IL-4-pretreated macrophages at a translational level. The expression of M1 or M2 marker proteins was analyzed by using flow cytometry (Figure 4(d)). Graphs show the upregulation of iNOS and Cd86 (markers for M1 polarization) and slight downregulation of Cd163 (a marker for M2 polarization). These results imply that PGNO-media may be able to limit M2-related gene transcription and elicit M1 macrophage polarization [22].

### 3.6. The Anticancer Effects of PGNO-Media Stimulated Macrophages In Vitro

**Figure 5:**
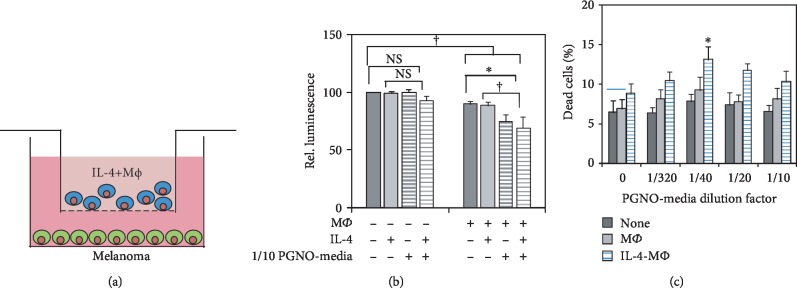
The anticancer effects of PGNO-media stimulated macrophages *in vitro*. (a) Illustrations showing indirect coculture of Raw 264.7 macrophages with B16F10 melanoma cancer cells. (b) Measurement of intracellular ATP of B16F10 cells with and without PGNO-media with coculture. 20 ng/mL IL-4 was pretreated to macrophage before 24 h of PGNO-media. (c) Flow cytometric measurement of PI-stained B16F10 cells at day 1 elapsed from coculture (^∗^*p* < 0.05). All the experiments were repeated three times. ^∗^*p* < 0.05; ^†^*p* < 0.01.

In order to test the anticancer effects of PGNO-media-activated macrophages, macrophages were cocultured in an indirect contact with B16F10 mouse melanoma cells as shown in Figure 5(a). B16F10 cells were cocultured with IL-4-pretreated or nontreated macrophages in a 0.4 *μ*m pore-sized transwell system. The cytotoxic immune actions of macrophages were analyzed by measuring intracellular ATP of melanoma cells after 24 h of cocultures (Figure 5(b)). The values were expressed relatively to monocultured control cells. The coculture of cancer with macrophage reduced the ATP values of cancer cells significantly in all cases. When PGNO-media was added, the reduction became more significant, especially when macrophages were pretreated with IL-4. It was confirmed by PI dye penetration assay that shows dead cell ratios as shown in Figure 5(c). The ratios of dead cells increased with addition of PGNO-media, especially when macrophages were pretreated with IL-4. This coculture study suggests that PGNO-media enhance the cytotoxicity of macrophages through activating secretion of cytokines.

### 3.7. The Anticancer Effects of PGNO-Media Stimulated Macrophages In Vivo

**Figure 6:**
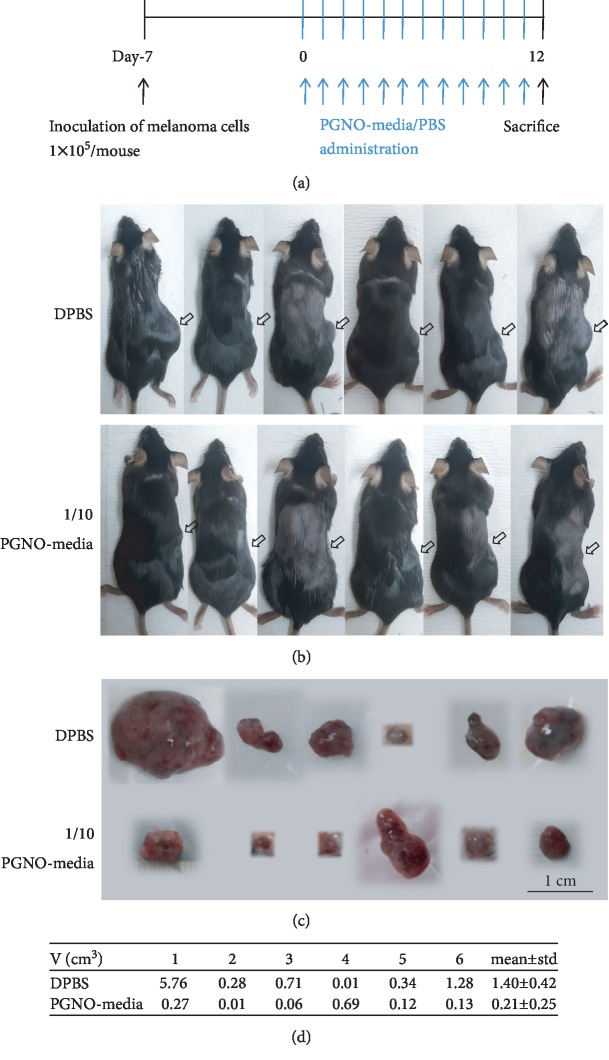
The anticancer effects of PGNO-media *in vivo*. (a) Simple scheme of animal experiment. (b) Pictures of 12 mice with melanoma syngeneic mice after 11 days of daily administration of DPBS or PGNO-media. Arrows mark the positions of tumor. (c) Pictures of tumor tissue from each mice at day 12. (d) A table showing the volume of tumor tissue from each mice and the average and standard deviation for the two groups.

The anticancer effects of PGNO-media were examined with a melanoma syngeneic model in mice.  Figure 6(a) shows a simple scheme of the animal experiment. Mice administered with DPBS were used as sham controls, and there were no dead mice during experiments for 19 days.  Figure 6(b) shows the 6 mice of the sham group and the 6 mice of the experimental group after daily administration of DPBS or PGNO-media near the melanoma for 12 days, respectively. Arrows in each picture indicate the positions of tumor. It is clearly evident that the sizes of tumors were significantly smaller with PGNO-media, in comparison with DPBS treatment.  Figure 6(c) shows the tumor tissues extracted from mice in the order shown in Figure 6(b). The width, length, and height of tumor tissues were measured, and Figure 6(d) summarizes the volumes in the table. The average value was smaller more than two times. When the biggest one in the DPBS group was removed, the difference was still more than two times. This shows the tendency of retarded growth of tumor by PGNO-media. Though we could not measure the exact growth rate of tumor from the beginning, photos taken at days 7, 10, and 11 that showed the growth rates of tumors as well as their sizes were quite different in two groups (Fig. [Supplementary-material supplementary-material-1] supporting information).

**Figure 7:**
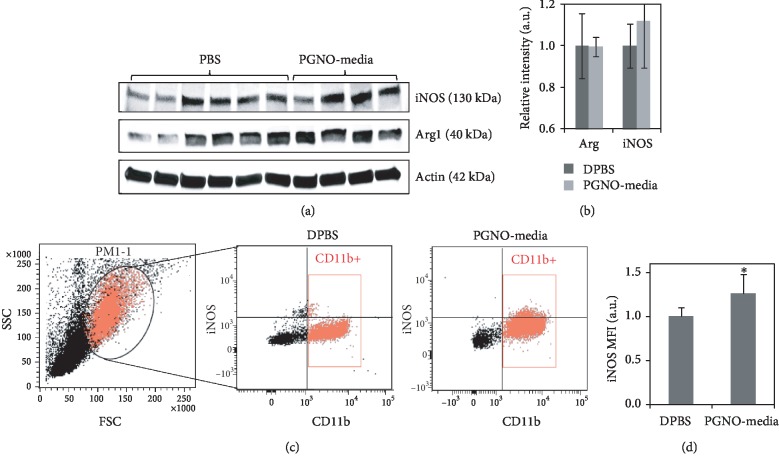
(a) The expression of iNOS and Arg1 protein in each tumor tissue. (b) The relative quantification of proteins to actin protein. (c) Flow cytometry data of cells harvested from mice peritoneal and immunostained with antibodies to iNOS and CD11b. Red boxed mark the CD11b+ cells. (d) The MFI of iNOS of the CD11b^+^ cells. ^∗^*p* < 0.05.

In order to evaluate the effect of PGNO-media on macrophage activation during tumor growth *in vivo*, we harvested tumor tissues and analyzed their expression of iNOS and Arg1. Due to the small sizes of some tissues, we used 6 samples from the PBS-treated group and 4 samples from the PGNO-media-treated group.  Figure 7(a) shows the western blot images, while Figure 7(b) shows the quantitative intensity values relative to actin protein. The protein expression levels were not significantly different from each other. Additionally, the peritoneal macrophages were harvested after sacrifice and stained with CD11b and iNOS antibodies.  Figure 7(c) shows the 2D plot of CD11b and iNOS stained peritoneal macrophages, where the CD11b-positive cells were gated with red squares.  Figure 7(d) shows the increased fluorescence of iNOS of CD11b+ cells from PGNO-media-administered mice. This data implies that when PGNO-media was injected during tumor growth, the peritoneal macrophages expressed more iNOS proteins.

## 4. Discussions

First of all, we successfully generated PGNO-media that contained RNS exclusively. Colorimetric analysis showed that our PGNO-media mainly contained RNS and did not contain ROS such as H_2_O_2_. Since our whole PGNO-generating system was isolated from atmospheric environment, PGNO-media components were solely controlled by inlet gases, N_2_ and O_2_ in this study. Hot reactive species were generated by focused microwave, but the primary species were cooled down through a cooling system to make more stable chemical species such as NO and NO_2_. Therefore, the chemical species that purged into DI water did not contain free electrons to generate OH^·^ or O/H atoms which possibly resulted in the generation of ROS. The expected chemical reactions were suggested in a previous report [31]. In addition, we guess those RNS have long lifetime, because PGNO-water was generated after purging water of dissolved oxygen elimination. The lifetime of NO^·^ in aqueous solution is about several min and decreases with an increase of the environmental O_2_ concentration [39–41]. The half-life time of NO in our PGNO-water was electrochemically measured for about 6 h, and NO was not completely perished after 16 h from PGNO generation [33]. To confirm the species measured by an electrochemical method in our PGNO-water, we tried other modalities such as EPR with MGC (N-(dithiocarbamoyl)-N-methyl-D-glucamine, sodium salt, Dojindo Molecular Technologies, Inc.) and spectroscopic analysis with hemoglobin molecules following a previous report [42]. We found radical forms in EPR measurement and hemoglobin oxidations in spectroscopy (data are not included here). However, the signals were different from a control experiment with well-known NO donor SNAP. Though the molecular form is not exactly understood yet, we can assume those reactive nitrogen radicals as well as NO_x_^−^ may react with biomolecules in the media to induce cascade processes in biological systems.

The effects of RNS on biological systems are known to depend on their concentrations [43, 44]. NO in low concentration can activate cell function initiating cGMP signaling pathways, but in high concentration, it can induce cell death, releasing cytochrome C from mitochondria [43, 44]. In a previous report, it was shown that NO radicals in PAW also increased cell growth in low concentration and became fungicidal in high concentration [45]. In the case of PGNO-media, we found that dilution in the range of 1/1280 to 1/20 is nontoxic and activates metabolic activity of macrophages. Even dilution of 1/10 did not induce apoptosis, though it reduced metabolic activity slightly. Figures 2 and 3 show that the potential of PGNO-media for macrophage activation can be enhanced when it was less diluted. Based on the *in vitro* data, we used 1/10 PGNO-media in coculture or animal studies, in order to add the metabolic toxicity on cancer cells, as well as to induce proinflammatory responses from M2-polarized macrophages. However, in a coculture study, we found that 1/10 PGNO-media did not reduce the metabolic activity of B16F10 cells at all. This implies that the sensitivity to PGNO-media is different according to cell type. Based on this cell study, we can hypothesize that the reduction of tumor size in mouse experiments was attributed to the modulation of immune systems rather than to the direct cytotoxicity of PGNO-media on melanoma cells. The results of western blot of tumor tissues and the flow cytometry analysis of peritoneal macrophages support that 1/10 PGNO-media can modulate whole body immune activities [46]. We speculate that RNS inside PGNO-media played roles in modulating the macrophage polarization.

Over decades, there have been many trials to inhibit the function of TAM and to convert them into M1-polarized cells [25]. Injecting NO^·^ donor NOC-18 or M1 activated macrophage to form a high concentration of NO/RNS in tumor tissues of a mouse renal cell carcinoma model, which could weaken tumor growth, but could not degenerate tumor [47, 48]. Macrophages separated from a malignant mesothelioma patient were M1 activated *in vitro* and transferred back to the host. Though a high level of TNF-*α* was produced in tumors and the tumor size was reduced, the injected macrophages were returned back to the M2-like mode in tumors by secreting anti-inflammatory cytokines and lipid mediators in the tumoral microenvironment [49–51]. Thus, maintaining the M1 activation of macrophages in tumor tissues was indicated as a solution.

Our experimental data support that PGNO-media stimulation can restrain the function of TAM-like macrophages and convert their function to M1-like macrophages. IL-4 is known to induce polarization of M2 by inducing downstream pathways such as a phosphorylation of STAT6 and an expression of IRF4 [35, 36]. Treatment of PGNO-media to IL-4-induced M2-like macrophages resulted in upregulation and downregulation of M1-and M2-related genes, respectably. In vivo treatment of PGNO-media also resulted in an inhibition of tumor growth and an upregulation of iNOS expression in peritoneal macrophages. Our study indicates that PGNO-media negatively regulates tumor-mediated manipulation of macrophages. In addition, it should be pointed that the action of PGNO-media on the macrophage activation and anticancer activity was not linearly dose dependent as metabolic activity and intracellular NO level were (Figures 2 and 3). The curves of the Cd86 transcription level (Figure 4(d)) as well as the tumor cytotoxicity level (Figure 5(c)) showed some optimal dose ranges for M2-like macrophage activation. This would give further information on these processes, as it points to a dual mode of action.

Our data proposes a new RNS donor that can maintain the M1-like activation of TAM. Since the PGNO-media is purely composed of RNS, there are no remaining chemicals or unexpected byproducts. Furthermore, there is a possibility that PGNO-media can enhance a whole body immune activity as shown in Figure 7. This can be helpful for the patients who suffer from the low immune activity due to anticancer drug administration. We believe that our experiments provide the possibility of PGNO-media application in anticancer therapy to retard tumor growth as well as activate innate immune activities. However, further studies using primary macrophages instead of Raw 264.7 cell line derived from leukaemic mice should be performed to confirm the action of PGNO-media in innate immune cells. And also, the tumor-associated macrophages instead of peritoneal macrophages should be investigated to understand the direct effects of PGNO-media on the microenvironment of tumors.

## Data Availability

The data used to support the findings of this study are available from the corresponding author upon request.

## Supplementary Materials

Supplementary Materials Figure S1: the original film image of Figure 4(c). In order to reduce the amount of antibodies, we cut the protein-transferred membrane at the position for the target protein size. Each cut membrane was reacted with specific antibodies and then incubated with chemiluminescence solution. They were arranged and fixed on a cassette. The X-ray film was reacted with them for four different times and then developed in a dark condition. (a) The original film image. The red colored rectangles were selected for the data. (b) The cut film image. (c) The band at 130 kDa was used for iNOS, and the band at 42 kDa was used for *β*-actin. Figure S2: the original film images of Figure 7(a). After staining with antibodies, the film images were acquired using a ChemiDoc machine (Bio-Rad). (a) The images acquired by the ChemiDoc. From left to right, the image is HRP chemiluminescence, colorimetric, and composition, respectably. (b) The red colored rectangles were selected for the data. Figure S3: daily measured weight and photos taken at day 7 and day 10 of experimental mice (a). The photos of mice taken at days 7, 10, and 11. Since we used normal mice with hair, it was difficult to measure tumor without sacrifice. To estimate roughly the tumor size, photos were taken with anesthesia. The sizes of tumor tissues were measured at the end of the experiment after sacrifice, and the data was put in the text. (b) The weight of mice was measured daily. Though we could not measure the size of tumor daily, we measured animal weight. Since it was performed without anesthesia, it had quite big errors. The units are gram (bar graph).
