# Supplementary material for: Anticancer Activity of Liquid Treated with Microwave Plasma-Generated Gas through Macrophage Activation
Source: Oxid Med Cell Longev. 2020 Jan 31;2020:2946820. doi: 10.1155/2020/2946820 (PMC7013299; doi:10.1155/2020/2946820)

Supporting Information

Anti-cancer activity of liquid treated with microwave plasma-generated gas through macrophage activation

[1,2†]Chae Bok Lee, [3†]Il Hwan Seo, [4]Myoung-Won Chae, [3]Jae Woo Park, [3,4]Eun Ha Choi, [5*]Han Sup Uhm, and [3*]Ku Youn Baik

[1]Department of Medical Science, and [2]Department of Microbiology, College of Medicine, Chungnam National University, Daejeon 35015, Republic of Korea, [3]Department of Electrical and Biological Physics, Kwangwoon University, Seoul 01897, Republic of Korea, [4]Plasma Bioscience Research Center, Kwangwoon University, Seoul 01897, Republic of Korea, [5]New Industry Convergence Technology R&D Center, Ajou University, Suwon 16499, Republic of Korea

[†]Both authors contributed equally.

[*]Corresponding author e-mail: hsuhm1970@gmail.com and kybaik@kw.ac.kr

Figure S1. The original film image of Figure 4(c).

In order to reduce the amount of antibodies, we cut the protein-transferred membrane at the position for the target protein size. Each cut membrane was reacted with specific antibodies and then incubated with chemiluminescence solution. They were arranged and fixed on a cassette. The X-ray film was reacted with them for four different times and then developed in a dark condition. (a) The original film image. The red colored rectangles were selected for the data. (b) The cut film image. (c) The band at 130 kDa was used for iNOS, and the band at 42 kDa was used for β-actin.

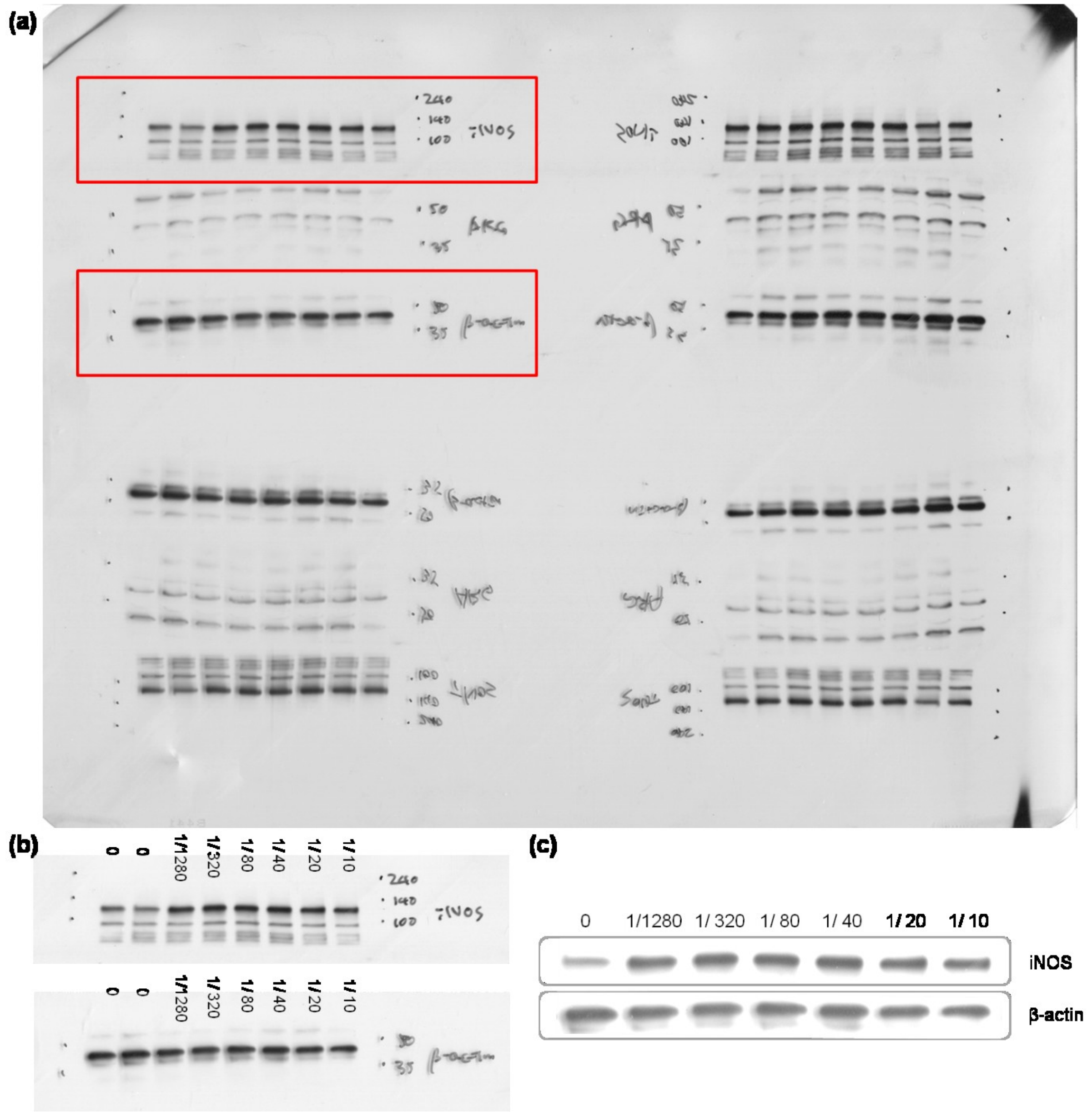

Figure S2. The original film images of Figure 7(a).

After staining with antibodies, the film images were acquired using ChemiDoc machine (BioRad). (a) The images acquired by the ChimiDoc. From left to right, image is HRP chemiluminescence, colorimetric, and composition, respectably. (b) The red colored rectangles were selected for the data.

(a)

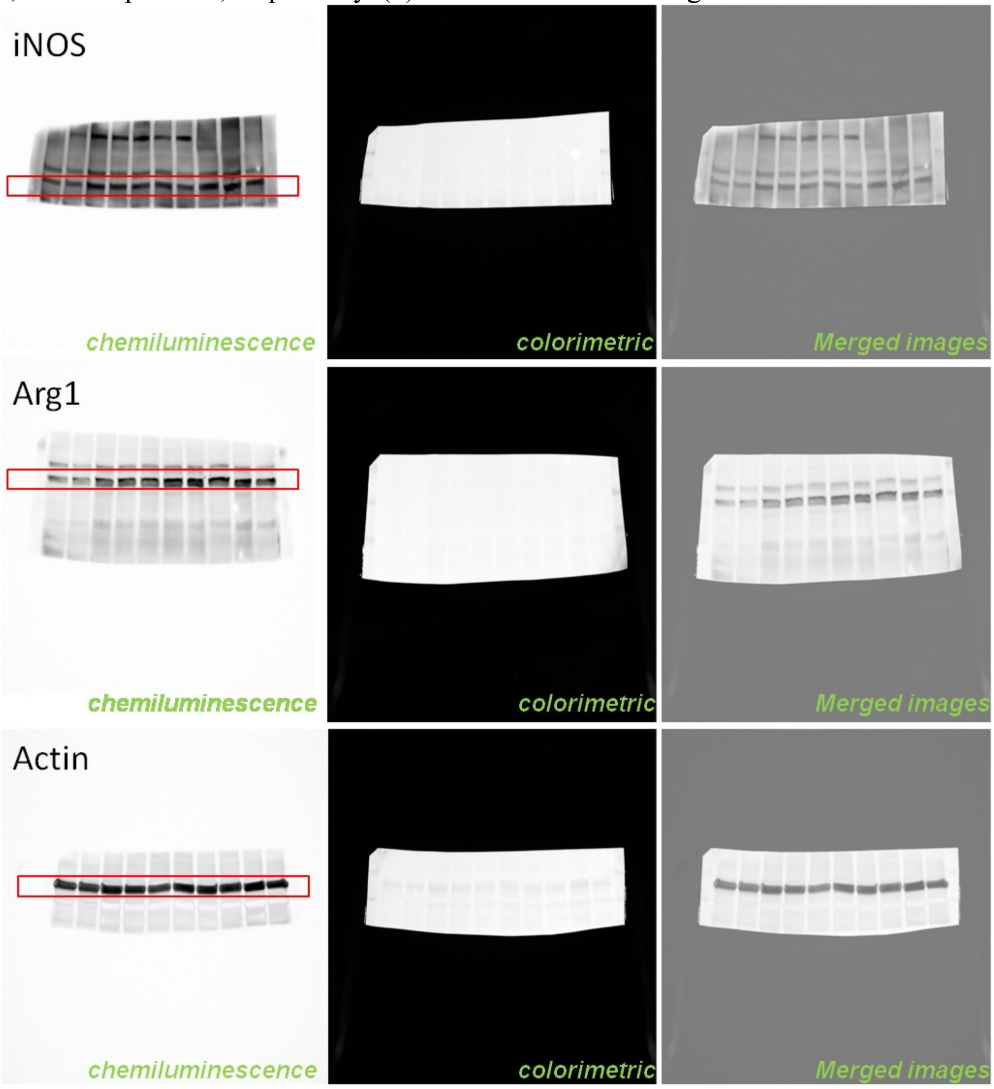

(b)

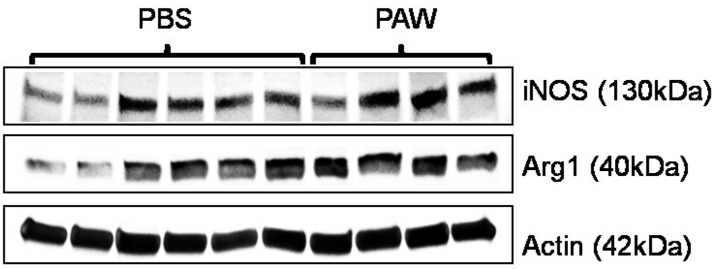

Figure S3. Daily measured weight and photos taken at day 7 and day 10 of experimental mice

(a) The photos of mice taken at day 7, 10, and 11. Since we used normal mice with hair, it was difficult to measure tumor without sacrifice. To estimate roughly the tumor size, photos were taken with anesthesia. The sizes of tumor tissues were measured at the end of the experiment after sacrifice, and the data was put in the text.

(b) The weight of mice measured daily. Though we could not measure the size of tumor daily, we measured animal weight. Since it was performed without anesthesia, it had quite big errors. The units are gram. The bar graph

(a)

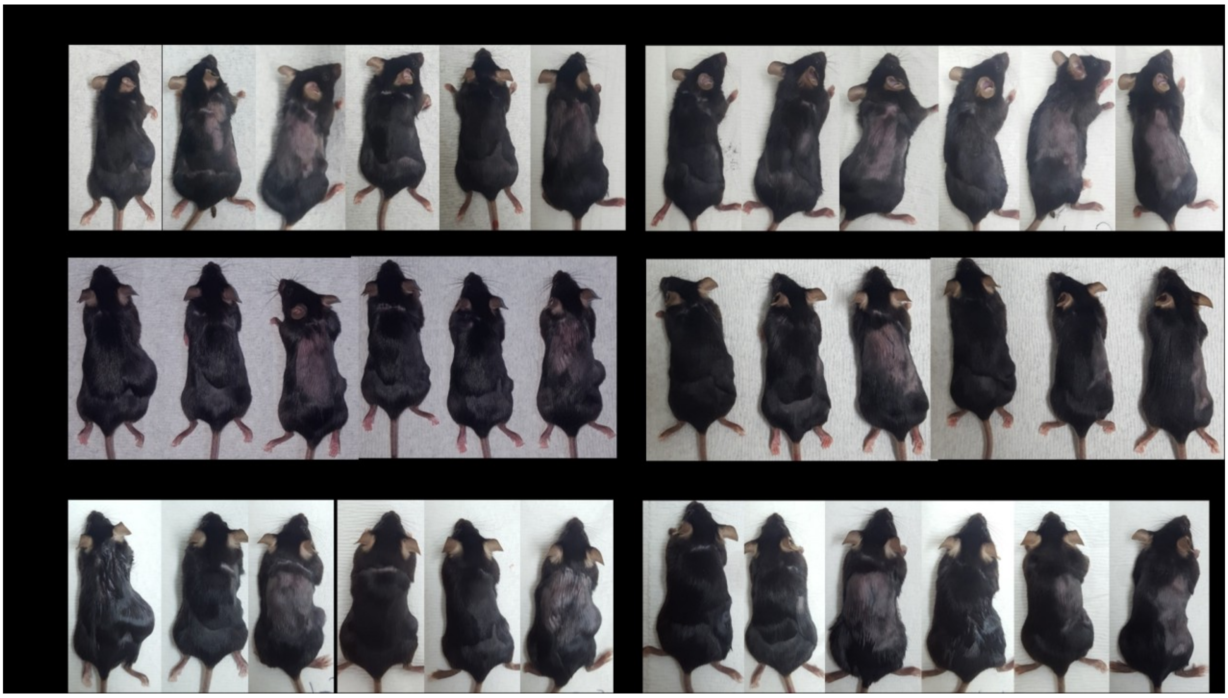

(b)

Cage	#	D0	D1	D2	D3	D4	D5	D6	D7	D8	D9	D10
1 (Sham)	1	24	24.1	23.4	23.7	23.3	23.4	23.7	25.1	25.3	25.6	25.9
	2	20.9	21	20.5	19.7	20.6	21	20.8	20.6	21	21.2	20.9
	3	21.2	21.6	21.6	22	21.8	21.1	21.3	21.4	22	22.2	21.2
2 (Sham)	1	21.2	21.5	20.8	21.3	21	21.2	21.1	21.3	21.1	21.4	21.3
	2	21.8	21.7	21.6	22.3	22.2	22	21.8	22.1	22.5	22.6	21.14
	3	21.8	22	22	22.4	21.9	21.5	21.4	22.2	22.3	22	21.95
3	1	21.9	22.2	22.6	22.7	22.8	22.2	22.5	22.6	23	22.8	22.5

(Exp)	2	21.6	22.2	22.4	22.9	22.4	22.3	22.4	23.1	23.3	23.2	22.43
	3	21.2	20.8	21	21.3	21.7	21.6	21.1	21.7	21.9	22.2	21.25
4 (Exp)	1	22.3	22.2	21.6	21.8	21.9	23	22.6	22.3	22.3	22.6	22.36
	2	21.6	22.1	21.2	21	21.4	21.9	22.7	22.1	21.8	21.9	21.99
	3	22.5	22.7	22	22.3	23.1	22.7	22.6	22.8	23.1	23.1	22.92

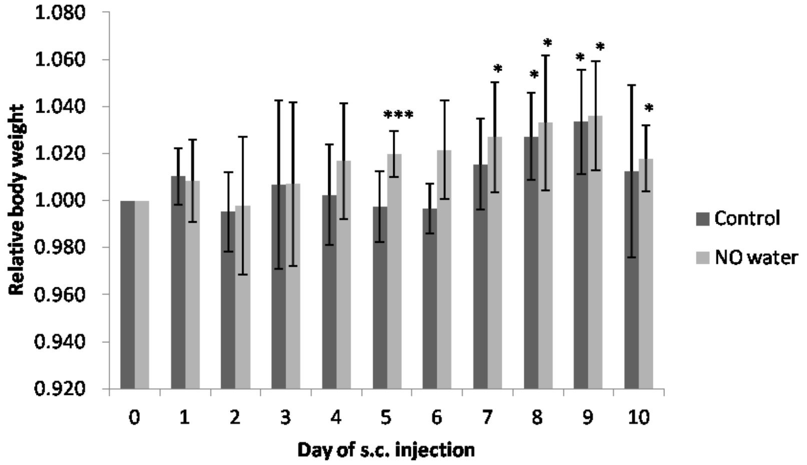

Supplement: Supplementary Materials — Figure S1: the original film image of Figure 4(c). In order to reduce the amount of antibodies, we cut the protein-transferred membrane at the position for the target protein size. Each cut membrane was reacted with specific antibodies and then incubated with chemiluminescence solution. They were arranged and fixed on a cassette. The X-ray film was reacted with them for four different times and then developed in a dark condition. (a) The original film image. The red colored rectangles were selected for the data. (b) The cut film image. (c) The band at 130 kDa was used for iNOS, and the band at 42 kDa was used for β-actin. Figure S2: the original film images of Figure 7(a). After staining with antibodies, the film images were acquired using a ChemiDoc machine (Bio-Rad). (a) The images acquired by the ChemiDoc. From left to right, the image is HRP chemiluminescence, colorimetric, and composition, respectably. (b) The red colored rectangles were selected for the data. Figure S3: daily measured weight and photos taken at day 7 and day 10 of experimental mice (a). The photos of mice taken at days 7, 10, and 11. Since we used normal mice with hair, it was difficult to measure tumor without sacrifice. To estimate roughly the tumor size, photos were taken with anesthesia. The sizes of tumor tissues were measured at the end of the experiment after sacrifice, and the data was put in the text. (b) The weight of mice was measured daily. Though we could not measure the size of tumor daily, we measured animal weight. Since it was performed without anesthesia, it had quite big errors. The units are gram (bar graph). [file 2946820.f1.pdf]
